# Mixture × Genotype Effects in Cereal/Legume Intercropping

**DOI:** 10.3389/fpls.2022.846720

**Published:** 2022-04-01

**Authors:** Dereje T. Demie, Thomas F. Döring, Maria R. Finckh, Wopke van der Werf, Jérôme Enjalbert, Sabine J. Seidel

**Affiliations:** ^1^Crop Science Group, Institute of Crop Science and Resource Conservation, University of Bonn, Bonn, Germany; ^2^Agroecology and Organic Farming Group, Institute of Crop Science and Resource Conservation, University of Bonn, Bonn, Germany; ^3^Department of Ecological Plant Protection, Faculty of Organic Agricultural Sciences, University of Kassel, Witzenhausen, Germany; ^4^Crop Systems Analysis Group, Wageningen University, Wageningen, Netherlands; ^5^Université Paris-Saclay, INRAE, CNRS, AgroParisTech, GQE-Le Moulon, Gif-sur-Yvette, France

**Keywords:** cultivar combination, intercropping performance, mixture, mixing ability, trait combination

## Abstract

Cropping system diversification through annual intercropping provides a pathway for agricultural production with reduced inputs of fertilizer and pesticides. While several studies have shown that intercrop performance depends on the genotypes used, the available evidence has not been synthesized in an overarching analysis. Here, we review the effects of genotypes in cereal/legume intercropping systems, showing how genotype choice affects mixture performance. Furthermore, we discuss the mechanisms underlying the interactions between genotype and cropping system (i.e., sole cropping vs. intercropping). Data from 69 articles fulfilling inclusion criteria were analyzed, out of which 35 articles reported land equivalent ratio (LER), yielding 262 LER data points to be extracted. The mean and median LER were 1.26 and 1.24, respectively. The extracted genotype × cropping system interaction effects on yield were reported in 71% out of 69 publications. Out of this, genotype × cropping system interaction effects were significant in 75%, of the studies, whereas 25% reported non-significant interactions. The remaining studies did not report the effects of genotype × cropping system. Phenological and morphological traits, such as differences in days to maturity, plant height, or growth habit, explained variations in the performance of mixtures with different genotypes. However, the relevant genotype traits were not described sufficiently in most of the studies to allow for a detailed analysis. A tendency toward higher intercropping performance with short cereal genotypes was observed. The results show the importance of genotype selection for better in cereal/legume intercropping. This study highlights the hitherto unrevealed aspects of genotype evaluation for intercropping systems that need to be tackled. Future research on genotype effects in intercropping should consider phenology, root growth, and soil nutrient and water acquisition timing, as well as the effects of weeds and diseases, to improve our understanding of how genotype combination and breeding may help to optimize intercropping systems.

## Introduction

In the past few decades, agricultural intensification has resulted in increased yields of pure line crops (Blomqvist et al., 2020); this has been accompanied by the simplification and homogenization of production systems and concentration on very few species as human diet staples (Khoury et al., 2014). Genetic uniformity and loss of diversity in the agricultural landscape (Hazell and Wood, 2008; Gregory and George, 2011) are characteristics of intensive agriculture, increasing vulnerability to climate change (Lin et al., 2008), and pathogen invasions (Anderson et al., 2004; Savary et al., 2019). Diversifying crop production systems is a promising pathway to tackle such vulnerabilities (Renard and Tilman, 2019; Hufnagel et al., 2020; Tscharntke et al., 2021). Diversification approaches can be classified into two categories: (1) integration of underutilized crops into the system; and (2) diversification of the production system through crop rotation, mixed cropping, and/or catch crops (Mustafa et al., 2019). More efficient utilization of resources with beneficial effects on the environment could also be gained by the integration of livestock with temporal and spatial crop diversification, such as forage legume intercropping with grain cereals (Danso-Abbeam et al., 2021). Crop diversification includes practices that significantly improve crop productivity, especially benefiting rural smallholders (Makate et al., 2016), and enhance overall ecosystem services without compromising crop yield (Tamburini et al., 2020; Beillouin et al., 2021; Ditzler et al., 2021). Annual intercropping is one form of cropping system diversification, which allows high productivity and reduction of fertilizer and pesticide input (Bedoussac et al., 2015; Li et al., 2020b) thereby substantially minimizing the negative environmental impacts of agriculture. Furthermore, crop diversification provides insurance against crop failure for farmers (Lithourgidis et al., 2011; Gaba et al., 2015).

Mixing crop species may be done with annual crops or perennial crops on a gradient of complexity from two to several species (Malézieux et al., 2009; Finckh and Wolfe, 2015). Cereal/legume intercropping systems are widely used across the world, particularly by smallholders, producing high-quality cereal and legume grains in an economically sustainable, environmentally friendly, and efficient way. Using legume crops in a mixture with cereals may significantly mitigate N_2_O fluxes derived from fertilizer, hence providing an effective way to reduce greenhouse gas emissions from cropping systems (Senbayram et al., 2015). Furthermore, intercropping was found to produce higher cereal protein concentration (Bedoussac and Justes, 2010; Timaeus et al., 2021b), higher grain yields (Yu et al., 2016), higher yield stability (Raseduzzaman and Jensen, 2017), and better abiotic and biotic stress resistance (Bedoussac et al., 2015; Timaeus et al., 2021a) than sole crops.

Intercropping performance is often measured by the land equivalent ratio (LER), an index measuring the relative land area required to produce the same yields (or any other services, such as biomass) in sole crops as obtained from a unit area of intercrop. An LER greater than one indicates that intercropping uses the land more efficiently than pure stands to produce the desired outputs (Mead and Willey, 1980).

Several studies have shown that the general performance of intercropping systems depends on the genotypes used in the mixture (e.g., Hauggaard-Nielsen and Jensen, 2001) and that the performance in a mixed stand can be poorly correlated to performance in a pure stand (Annicchiarico et al., 2019). Different genotypes of legumes may have different responses in terms of phenology and morphology (Annicchiarico and Filippi, 2007) when compared in sole crops vs. mixtures. Hence, a specific selection of genotypes for intercropping is important (Giles et al., 2017), and intercrop yield advantage could be achieved by selecting specific traits of both species (Berghuijs et al., 2020). Therefore, it has been suggested that specific breeding of genotypes for intercropping is needed to improve complementarity of the intercropping partners (Annicchiarico et al., 2019; Haug et al., 2021).

Cereal/legume mixtures could include systems where both species have similar phenology but contrasting morphology, or, alternatively, contrasting phenology and morphology, resulting in temporal and/or spatial niche complementarity (Gaudio et al., 2019). The ecological niche separation concept posits that the different species involved may have different resource requirements at different times, as well as for different sources of nutrition (Malézieux et al., 2009). In addition to niche complementarity, intercrop performance can also be due to additional ecological mechanisms (Loreau and Hector, 2001). Facilitation effects may exist between mixed species, such as synergy in the use of phosphorus (Hinsinger et al., 2011; Li et al., 2020). The species complementarity effect, which measures the overall shift of relative yields in mixtures vs. sole crop, has a higher effect on yield gain than the selection effect, which defines how these shifts in relative yields are correlated to sole crop yields (Li et al., 2020a). Complementarity is a paramount feature in cereal/legume intercrops grown under low-nitrogen (N) conditions, in which biological N fixation by the legume and strong competition for soil-N by the cereal may synergize to enhance yield and grain quality.

Choosing plant genotypes for specific intercropping systems is, however, laborious and costly, if only because assessing intercropping performance also requires the inclusion of sole crops in field experiments for comparison and estimation of the benefits of mixing. Testing genotypes in mixtures easily results in a curse of dimensionality. For instance, with five genotypes of a cereal and five genotypes of a mixture, 25 mixtures should be tested along with 10 pure stands. Optimal species traits likely depend on the companion species, such that all possible combinations are preferably tested. Note that incomplete designs have been proposed to deal with this challenge of dimensionality (Hinsinger et al., 2011), and shown to be efficient to estimate mixing abilities (Haug et al., 2021). Testers and reciprocal breeding schemes have been proposed to co-breed species (Sampoux et al., 2020). Recent technologies, such as genomic selection strategies, could help select traits for breeding for intercropping accurately (Bančič et al., 2021). However, better knowledge on genotypes and their associated trait effects in intercropping is needed to make selection more targeted.

General and specific mixing ability of genotypes of single species has been studied to determine contrasting traits in sole cropping and in mixtures, and the theoretical background has been discussed with respect to species mixtures (Wright, 1985). Historically, multiple studies have evaluated different crop genotypes for complementarity in intercropping (Francis et al., 1976; Smith, 1985; Smith and Zobel, 1991; Davis and Woolley, 1993). Abundant research has been conducted, but the knowledge on genotype effects in intercropping is fragmented and has not been compiled to deliver necessary knowledge for designing optimized intercropping systems. Here, we aim to provide a current update by linking recent advances through a review. In particular, we address the knowledge gap concerning the mechanisms involved in genotype × cropping system interaction. This review is intended to answer the following questions: (i) How do different genotypes and/or traits of a species in cereal/legume intercropping systems affect the performance of the mixture? (ii) What are the mechanisms underlying the interaction of the genotypes in the intercropping system? and (iii) What are the current knowledge gaps in genotype evaluation for intercropping systems?

## Materials and Methods

### Literature Search and Publication Screening

We conducted a systematic map, using the science databases Web of Science, Scopus, Science Direct, and Google Scholar. Keywords used for searching suitable publications were “genotype interaction in inter/mixed cropping system” OR “cultivars interaction in inter/mixed cropping system” OR “varieties interaction in inter/mixed cropping system” OR “cereals in inter/mixed cropping system” and scientific names (genus and species name) and common names of cereals species with intercropping and mixed cropping. The slash (/) was not used in a search; here, it is used for simplified expression of search terms (i.e., intercropping OR mixed cropping). A full list of the search terms is given in the Supplementary Table 1. In addition, secondary literature cited in selected articles were also looked up and included if relevant. The latest search was conducted on 12 April 2021.

To select the relevant articles, we used the following inclusion criteria: (i) studies from cereal/legume intercropping with both grain and forage legumes, (ii) studies evaluated at least two genotypes of at least one of the mixed species, (iii) peer-reviewed full-length articles published in English, (iv) studies reporting original research data, and (v) only field experiments, excluding greenhouse or pot experiments. No restriction was made against the type of mixture design, e.g., with respect to plant density, such as additive, replacement (substitution), or intermediate design. The information extracted from the original research articles was categorized in a digital database and analyzed following the Preferred Reporting Items for Systematic Review and Meta-Analyses (PRISMA) guidelines (Moher et al., 2009).

### Variables and Data Extraction

Data on genotype performance originated from different management and different zones, resulting in large differences in yield. Hence, an index was necessary to characterize the performance of genotypes in intercropping in relation to their respective pure stands (Mead and Willey, 1980). We used the LER (Equation 1) as a key metric to measure intercrop yield advantage (or disadvantage) by reference to the pure crop yields of mixed genotypes. We also retrieved the results of any ANOVA analyzing genotype and cropping system main effects and their interaction. Furthermore, individual studies were scrutinized by assessing conclusions and interpretations about the effects of different traits (phenology and morphology) of species in mixtures to identify the general mechanisms responsible for cereal/legume intercropping yield advantage.

Different variables were extracted from each study (Table 1) in the core set of publications. Information, like intercropping design (design of the mixing system, i.e., substitutive or additive or intermediate), country of the experiment, number of genotypes, and other related variables, was extracted from each publication. Significance (or non-significance) of “genotype” effect, “cropping system” effect (pure vs. mixed stand), and “genotype” × “cropping system” interaction effect on yield data was extracted from ANOVA tables of the articles. This was done by extracting results from the ANOVA of each article; any differences among articles regarding the structure of statistical analysis (e.g., fixed vs. random effects) were disregarded. The mechanisms of intercropping performance were extracted from the description of results, and the full article was consulted if needed. Some studies reported various types of mixtures, from different species of either cereals or legumes. In addition, in these cases, data were extracted from all combinations in which at least two genotypes of at least one of the partners were evaluated.

**TABLE 1:** Variables extracted from different studies.

Variables	Definition	Data type/Units
Title	Title of the publication	Text
Authors	Authors in publication	Text
Year	Publication year	Text
Journal	The journal in which the article was published	Text
Country	The country where the experiment was conducted	Text
Precipitation	The total rainfall during the growing period	Numerical
Soil texture	The texture of the soil in the experimental area	Categorical
Species and genotypes	The names of species and genotypes used in the experiment	Text
Number of genotypes	The number of genotypes of each species studied in the experiment	Numerical
Design	Plant density (additive/replacement/intermediate)	Categorical
Response variable	The response variable investigated	Text
Replication	How many times the treatment was replicated	Numerical
Number of locations	Number of the site where the experiment was conducted	Numerical
Number of seasons	Number of seasons during which the experiments were conducted	Numerical
Genotype, cropping system, and interaction effects	The statistical significance of interaction, cropping system, and genotype effect	Categorical
Interaction traits	List of traits/mechanisms highlighted as causal in crop interactions and intercropping performance	Categorical
LER	Land equivalent ratio	Numerical

The LER (Equation 1) of each genotype combination was extracted from the subset of articles reporting them, either directly when represented numerically, or in figures. Data from figures were digitalized using a web-based plot digitizer (Rohatgi, 2020), an online system used to extract data from images efficiently and accurately (Burda et al., 2017; Cramond et al., 2019). LER was reported in figures only in five articles (Rao and Willey, 1983; Odo, 1991; Watiki et al., 1993; Kontturi et al., 2011; Pappa et al., 2012; Barillot et al., 2014). The majority of the studies reported mean LER per genotype combination across multiple environments. However, in some cases, the studies reported data individually from each environment. If the mean LER across different environments was not reported, this mean was computed for each genotype combination of the species in the intercrop from the individual environments. When a study reported only the partial land equivalent ratio (PLER), the total LER was calculated for each genotype combination of the species in intercropping by summing the PLERs:


$${\text{LER}}_{\text{c}+\text{l}}={\text{PLER}}_{\text{c}/\text{l}}+{\text{PLER}}_{\text{l}/\text{c}}$$

where LER_c+l_ is the LER of the cereal genotype c with the legume genotype l; and PLER_c/l_ is the partial LER of genotype c in mixture with legume genotype l (and reciprocally for PLER_l/c_). This genotype combination-specific LER was used in further analysis. If neither LER nor PLER was reported, LER for each genotype in a given cereal–legume combination was calculated from yields in mono-cropping and intercropping.

When other treatments were applied (such as different row spacing, and sowing density or proportion), LERs were extracted or calculated from only one treatment. If different levels of N were used, data for each level of fertilizer were considered and averages computed for each genotype combination. In one study, results from two species of cereals or legumes were reported. Thus, data were recorded from each genotype combination from each species and analyzed. Therefore, at least 2 data points from each article (depending on number of genotypes of cereals and legumes) were extracted. In this way, we obtained 262 LER data points.

Since only few (10%) LER data points were reported from forage legume species combinations with cereals (2 articles with oats, 1 article with finger millet, and 2 articles with maize) all data from forage and grain legumes were combined and analyzed together.

### Data Analysis

The main effects of genotype and intercropping and their interaction effects were assessed by counting and calculating the proportion of articles that reported significant or non-significant effects on yields. In addition to the analysis of LER, a fixed-effects ANOVA model was used to test the effect of cereal species, design, and interaction effect on LER across cereal species by categorizing the dataset by cereal species. Because the number of data points of wheat was low (*n* = 5), and data records from barley and rice were only from replacement design, we excluded these three from the analysis. The number of data records per cereal species varied from 25 (finger millet) to 131 (maize). Similarly, a fixed-effects ANOVA model was used to test the effect of legume species, design, and interaction on LER across legume species by categorizing the dataset by legume species. However, faba bean, grass pea, guar and hairy vetch, berseem clover, and bitter vetch were excluded because the number of data points (two to four) was low. The mean comparison was done by Tukey’s honestly significant difference (HSD) test.

To assess the potential of genotype choice for optimizing LER, we calculated three indices using the extracted data from the articles (averages across the site years); to obtain these indices, we first calculated the maximum, median, and minimum LER across different genotype combinations for each article. Then (i) the difference between maximum and median LER was used as a measure for the potential of combined genotype choice to improve LER in comparison to a random choice; similarly, (ii) the difference between minimum and median LER was taken as a measure for the risk to choose an inappropriate genotype combination in comparison to a random choice; and (iii) the range, i.e., the difference between maximum and minimum LER from an article was used to characterize the maximum genotype combination effect within a study. The median used to calculate all three statistics were calculated from each individual article. The three statistics are equivalent when only two genotypes were evaluated. Because of sampling effects, it is expected that all three differences would tend to increase (in absolute terms) with increasing number of genotype combinations tested within a study (Schwarz, 2011); therefore, we plotted the indices against the number of genotype combinations. The extracted LER data were subjected to descriptive statistics; all analyses were conducted with R (R CoreTeam, 2020), and figures were produced using the R package ggplot2 (Wickham, 2016).

## Results

### Geographical Distribution and Characteristics of Studies

From about 4,000 search hits using all search terms, only 69 articles fulfilled the inclusion criteria (Table 2). The reported research studies were conducted in 28 different countries (Supplementary Table 2). The majority of data came from Africa (37%) followed by Europe (24%) and Asia (18%). The included studies considered different contrasting characteristics of genotypes of cereals and legumes evaluated.

**TABLE 2:** List of cereal and legume species in the 69 selected studies investigating genotype effects in intercropping; because some studies tested more than two species, the sum of studies across all crop species (152) is greater than 2 × 69 = 138.

Common name	Scientific name	No. of studies
**Cereals**		
Maize	*Zea mays*	30
Oat	*Avena sativa*	8
Wheat	*Triticum aestivum*	8
Finger millet	*Eleusine coracana*	6
Sorghum	*Sorghum bicolor*	6
Barley	*Hordeum vulgare*	5
Rice	*Oryza sativa*	5
Naked oat	*Avena nuda*	1
Durum wheat	*Triticum durum*	1
**Legumes**		
Common bean	*Phaseolus vulgaris*	17
Cowpea	*Vigna unguiculata*	13
Soybean	*Glycine max*	8
Pigeon pea	*Cajanus cajan*	7
Pea	*Pisum sativum*	7
Faba bean	*Vicia faba*	7
Berseem clover	*Trifolium alexandrinum*	5
Groundnut	*Arachis hypogaea*	3
White clover	*Trifolium repens*	2
Bitter vetch	*Vicia ervilia*	2
Common vetch	*Vicia sativa*	2
Hairy vetch	*Vicia villosa*	2
Guar	*Cyamopsis tetragonoloba*	1
Grass pea	*Lathyrus sativus*	1
Snail clover	*Medicago truncatula*	1
Serradella	*Ornithopus sativus*	1
Runner bean	*Phaseolus coccineus*	1
Caribbean stylo	*Stylosanthes hamata*	1
Subterranean clover	*Trifolium subterraneum*	1

**FIGURE 1:**
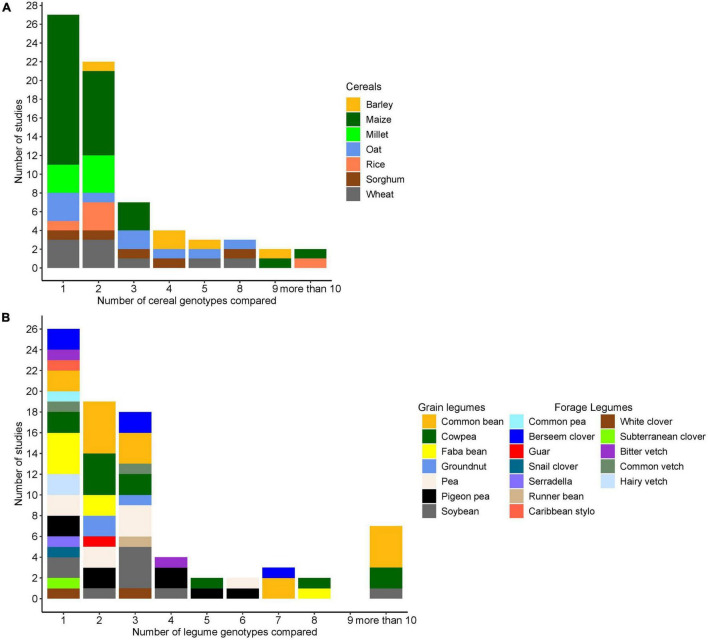
**(A)** Number of cereal genotypes evaluated in combination with legume species (each combination was categorized based on the cereal species). **(B)** Number of legume genotypes evaluated in combination with cereal species (each combination was categorized based on the legumes species). In both cases, if one genotype of one partner is evaluated, the other partner had at least 2 genotypes.

Overall, 9 cereal crop species and 19 legume species were evaluated in 69 publications with maize as the most frequently evaluated cereal species followed by oat and wheat. Common bean was the most frequently evaluated legume followed by cowpea and soybean. In the considered studies, common bean was only intercropped with maize. A single genotype was used in 62% of the studies for one of the partner species, i.e., in these studies, genotypic variation was only investigated in the other partner. On average, 4 cereal genotypes or 3 legume genotypes were compared per study, when excluding the single genotype studies (Figure 1). The most diverse comparison included 8 genotypes of cereal (*Avena sativa*) and 7 genotypes of legume species (*Trifolium alexandrinum)*, in a total of 56 cereal–clover combinations.

The majority of studies (55) evaluated grain legumes, whereas eight studies evaluated forage legumes, and a small proportion (6) of studies evaluated both forage and grain legumes together. The number of genotypes used in the studies varied, with similar numbers of studies reporting on (i) combinations of two or more cereal genotypes with two or more legume genotypes, (ii) one cereal genotype combined with two or more legume genotypes; or (iii) one legume genotype combined with two or more cereal genotypes (Table 3).

**TABLE 3:** Number of studies with one or more than one genotype of cereal and/or legume (*not included in this review) from 69 studies.

	1 cereal genotype	> 1 cereal genotype
1 legume genotype	*	16
> 1 legume genotype	27	27

*One article evaluated two cereal species resulting in a total of 70 datasets (out of one publication, two datasets were extracted).*

### Effect of Cropping System and Genotypes of Cereal/Legume on Intercropping Performance

#### Genotype × Cropping System Interaction

The extracted genotype × cropping system interaction effects on yield were reported in 49 (71%) studies out of 69 publications. Out of this, genotype × cropping system interaction effects were significant in 37 (75%) of the studies, whereas 12 (25%) of the studies reported non-significant interactions. The remaining studies did not report the effects of genotype × cropping system. In addition, intercropping main effects were reported in 38 (55%) studies. Out of this, the effect was significant in 27 (71%) and non-significant in 11 (29%) of the publications. Genotype main effects were reported in 37 (53%) studies; out of this, the genotype effect was significant in 25 (67%) and non-significant in 12 (33%) of the publications. The remaining studies did not mention the effects of cropping system and genotype effects.

#### Land Equivalent Ratio as Metric to Gauge Yield Advantage of Genotypes in Intercropping

From the 69 studies used for data extraction, 35 studies yielded 36 datasets (one study used two cereal species) and either reported the LERs directly or allowed calculation from the reported yield data. From these 36 datasets, 262 data points (cereal/legume genotype combinations) were extracted, based on a total of 85 cereal and 126 legume genotypes, with a number of cereal/legume combinations (LER) ranging from 2 to 22 per study.

**FIGURE 2:**
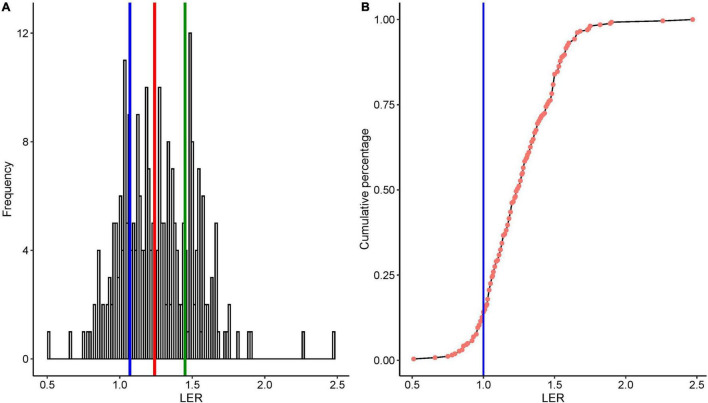
**(A)** Frequency distribution of LER from 35 studies, quartiles marked by blue and green; median marked by red-colored vertical lines; **(B)** cumulative percentage distribution of LER from 35 studies, 36 (datasets); the vertical blue line in panel **(B)** shows LER = 1.

**FIGURE 3:**
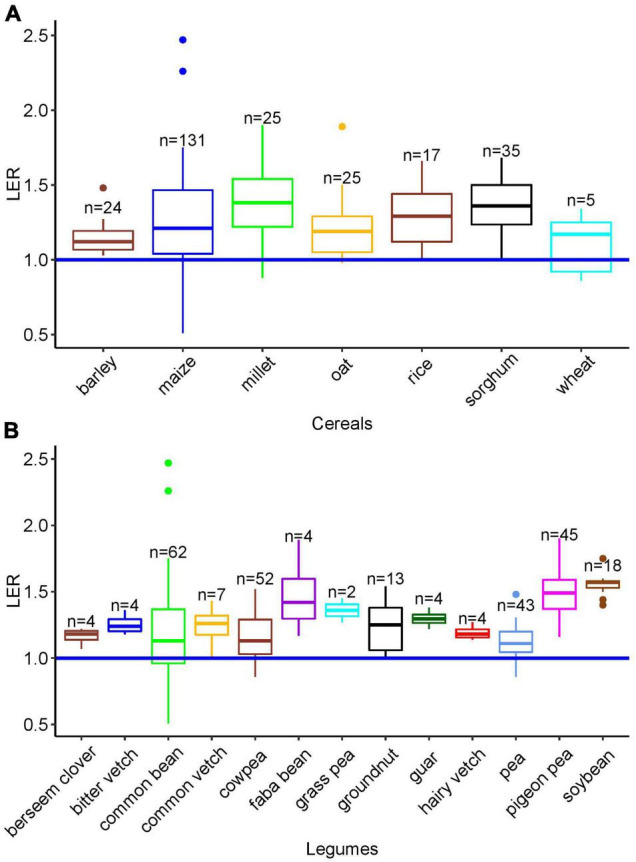
**(A)** LER of intercropping systems with different cereal components. **(B)** LER of intercropping systems with different legume components. Extracted from 35 studies with median (horizontal line), upper and lower quartiles (boxes), and 1.5 interquartile range (IQR) (whiskers). The horizontal blue line was drawn at LER = 1; *n*: number of data points. Although wheat, faba bean, berseem clover, bitter vetch, hairy vetch, and guar data were excluded from the ANOVA (*n* < 5), the data are shown in this graph for comparison.

The calculated mean and the median LER were 1.26 and 1.24, respectively (Figure 2), and LER was greater than 1.0 in 85% of the single cases. Although the number of data points for some cereals, especially wheat, may not be sufficient to compare the median LER with other cereals, the overall outcome was robustly > 1 with the highest median LER of 1.38 (*n* = 25) found in finger millet. The strikingly high variation in maize is in part due to the number of studies. In barley-based cropping systems, all of the LER data were greater than 1 (*n* = 22, range 1.05–1.48) (Figure 3).

**FIGURE 4:**
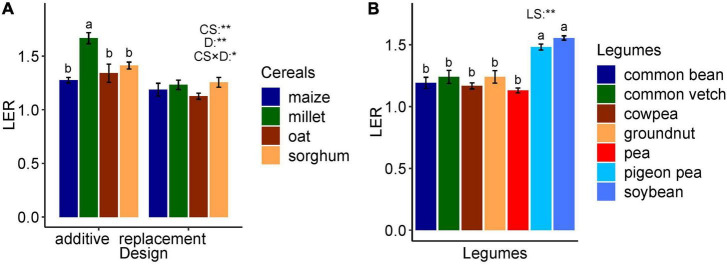
**(A)** Effect of cereal species and design on LER. **(B)** Effect of legume species on LER. The letters show the statistical differences between species. CS, cereals species; D, design; CS × D, species interaction with design; LS, legumes species; *significant (*p* < 0.05), **highly significant (*p* < 0.01), and the error bar is the standard error of the mean. The two designs (additive and replacement) are not represented for legumes because the effect of design is not significant.

The ANOVA resulted in highly significant differences across cereal species and design (*p* < 0.01). In addition, the interaction effect was significant (*p* < 0.05). The pairwise means comparison revealed that finger millet reached higher LERs in additive designs as compared to replacement designs, whereas no effect of design was found in maize and sorghum (see Supplementary Table 3 for ANOVA and Figure 4A). The ANOVA, across legume species and design, resulted in highly significant differences across legume species with pigeon pea and soybean exceeding other species but non-significant effects of design and interaction effect (*p* > 0.05) (see Supplementary Table 4 for ANOVA) (Figure 4B).

#### The Potential of Genotype Choice for Intercropping

**FIGURE 5:**
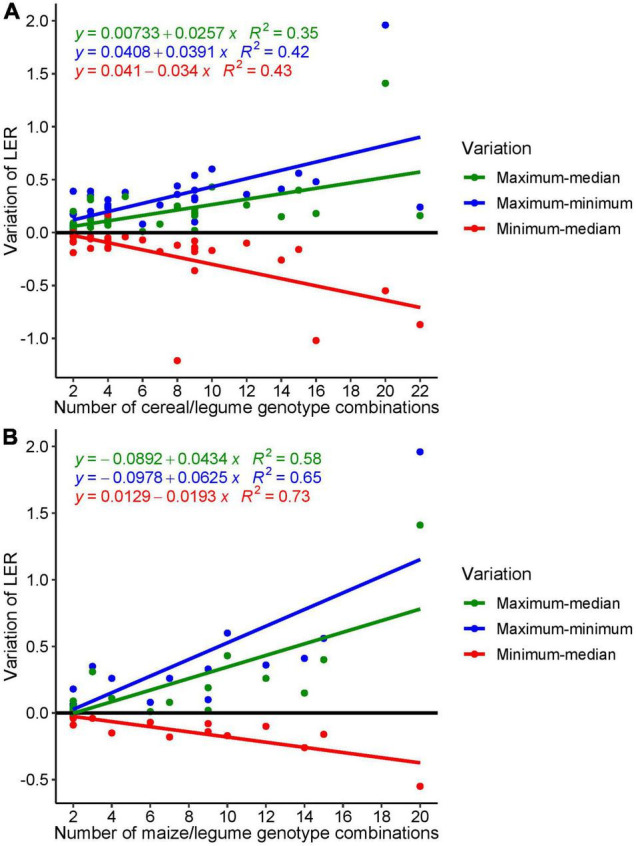
Variation of extracted LER: **(A)** all data points from cereal/legume intercropping extracted from 35 studies and **(B)** LER variation from maize-based intercropping extracted from 16 studies.

The distribution of the LERs within the studies around the median (Figure 5) indicates that genotype-specific effects play a role in the performance of mixtures in comparison to sole crops. Overall, the range (i.e., difference between maximum LER and minimum LER within a study) varied between 0 and 1.98, showing the potential of large genotype effects in intercropping. Conversely, there was a risk to obtain low LERs by non-appropriate genotype choice (i.e., as indicated by the difference of minimum LER and median LER, red points in Figure 5); the difference between minimum and median ranged from − 0.55 to 0. The largest LER range (1.96) was found in a study with 20 different genotypes combinations (10 bean and two maize genotypes) (Santalla et al., 2001); in the only other study with 20 genotypes combination (Hauggaard-Nielsen and Jensen, 2001), the range was 0.27, i.e., quite moderate (Table 5).

**TABLE 4:** Number of studies reporting significant and non-significant genotype, cropping system, and interaction effects, categorized by cereals.

	Cropping system effect			Genotype effect			Interaction effect		
Cereal	sig.	n.s.	n.r.	sig.	n.s.	n.r.	sig.	n.s.	n.r.
Barley	2	1	2	2	1	2	2	1	2
Maize	12	8	9	12	9	9	19	7	4
Millet	2	0	5	1	0	5	5	0	1
Oat	5	0	4	3	0	6	6	0	3
Rice	1	0	4	2	0	3	2	0	3
Sorghum	1	0	4	2	0	3	1	0	4
Wheat	4	2	3	3	2	4	2	4	3
Total	27	11	31	25	12	32	37	12	20

*sig., significant; n.s., not significant; n.r., not reported.*

**TABLE 5:** Deviations of LER from the median in cereal/legume intercropping extracted from 35 studies including between 2 and 20 mixtures, i.e., different genotype combinations (N).

Author	Cereal species	Legume species	No. cereal genotypes	No. legume genotypes	Range (max–min)	Max–median	Min −median	Design	(N)
Klimek-Kopyra et al., 2015	*Avena nuda*	*Vicia faba*	1	2	0.39	0.2	−0.19	add	2
Li et al., 2020	*Avena sativa*	*Vicia sativa*	1	3	0.2	0.05	−0.15	add	3
Baxevanos et al., 2017	*Avena sativa*	*Pisum sativum*	3	3	0.31	0.16	−0.15	repl	9
Ross et al., 2004	*Avena sativa*	*Pisum sativum*	2	2	0.25	0.11	−0.14	repl	4
Kontturi et al., 2011	*Avena sativa*	*Pisum sativum*	1	3	0.16	0.12	−0.04	add	3
Baxevanos et al., 2021	*Avena sativa*	*Vicia sativa*	4	1	0.17	0.11	−0.06	repl	4
Pappa et al., 2012	*Hordeum vulgare*	*Pisum sativum*	2	1	0	0	0	repl	2
Hauggaard-Nielsen and Jensen, 2001	*Hordeum vulgare*	*Pisum sativum*	4	6	0.24	0.16	−0.87	repl	22
Sanou et al., 2016	*Eleusine coracana*	*Vigna unguiculata*	2	2	0.13	0.08	−0.05	repl	4
Reddy et al., 1990	*Eleusine coracana*	*Vigna unguiculata*	3	3	0.4	0.22	−0.18	repl	9
Yadav and Yadav, 2001	*Eleusine coracana*	*Cyamopsis tetragonoloba*	2	2	0.16	0.085	−0.075	repl	4
Rao and Willey, 1983	*Eleusine coracana*	*Cajanus cajan*	2	4	0.44	0.25	−1.21	add	8
Ramakrishna and Ong, 1994	*Oryza sativa*	*Vigna unguiculata*	2	2	0.23	0.13	−0.09	repl	4
		*Arachis hypogaea*		2	0.13	0.07	−0.06	repl	4
		*Cajanus cajan*		2	0.31	0.13	0.17	repl	4
Rahlakrishna et al., 1992	*Oryza sativa*	*Cajanus cajan*	1	5	0.38	0.34	−0.04	repl	5
Tefera and Tana, 2002	*Sorghum bicolor*	*Arachis hypogaea*	3	3	0.54	0.18	−0.36	add	9
Odo, 1991	*Sorghum bicolor*	*Vigna unguiculata*	2	1	0.17	0.08	−0.09	repl	2
de Queiroz et al., 1988	*Sorghum bicolor*	*Vigna unguiculata*	8	1	0.36	0.24	−0.12	repl	8
Rao and Willey, 1983	*Sorghum bicolor*	*Cajanus cajan*	4	4	0.48	0.18	−1.02	add	16
Barillot et al., 2014	*Triticum aestivum*	*Pisum sativum*	1	3	0.39	0.33	−0.06	repl	3
Haymes and Lee, 1999	*Triticum aestivum*	*Vicia faba*	2	1	0.17	0.09	−0.08	add	2
Egbe et al., 2010	*Zea mays*	*Vigna unguiculata*	1	10	0.6	0.43	−0.17	repl	10
Watiki et al., 1993	*Zea mays*	*Vigna unguiculata*	1	15	0.56	0.4	−0.16	add	15
Goshime et al., 2020	*Zea mays*	*Phaseolus vulgaris*	9	1	0.33	0.19	−0.14	add	9
Gebeyehu et al., 2006	*Zea mays*	*Phaseolus vulgaris*	2	7	0.41	0.15	−0.26	add	14
Javanmard et al., 2020	*Zea mays*	*Lathyrus sativus*,	2	1	0.06	0.03	−0.03	add	2
		*Vicia villosa*		1	0.09	0.04	−0.04	add	2
		*Vicia ervilia*,		1	0.04	0.02	−0.02	add	2
		*Trifolium alexandrinum*		1	0.05	0.02	−0.02	add	2
Molatudi, 2012	*Zea mays*	*Phaseolus vulgaris*	1	2	0.18	0.09	−0.09	add	2
Pierre et al., 2017	*Zea mays*	*Glycine max*	1	3	0.35	0.31	−0.04	add	3
Zaeem et al., 2019	*Zea mays*	*Glycine max*	2	3	0.08	0.01	−0.07	add	6
Yang et al., 2018	*Zea mays*	*Glycine max*	3	3	0.1	0.02	−0.08	add	9
Javanmard et al., 2009	*Zea mays*	*Vicia ervilia*	2	1	0.07	0.03	−0.03	add	2
		*Trifolium alexandrinum*		1	0.04	0.02	−0.02	add	2
		*Vicia villosa*		1	0	0	0	add	2
		*Phaseolus vulgaris*		1	0.07	0.03	−0.03	add	2
Tamado et al., 2007	*Zea mays*	*Phaseolus vulgaris*	1	7	0.26	0.08	−0.18	add	7
Nassary et al., 2020a	*Zea mays*	*Phaseolus vulgaris*	1	2	0.09	0.05	−0.04	add	2
Muraya et al., 2006	*Zea mays*	*Phaseolus vulgaris*	2	2	0.26	0.11	−0.15	add	4
Dasbak and Asiegbu, 2009	*Zea mays*	*Cajanus cajan*	2	6	0.36	0.26	−0.1	add	12
Santalla et al., 2001	*Zea mays*	*Phaseolus vulgaris*	2	10	1.96	1.41	−0.55	repl	20
Nassary et al., 2020b	*Zea mays*	*Phaseolus vulgaris*	1	2	0.07	0.03	0.03	add	2

*In some studies, more than one legume species was evaluated; add, additive; repl, replacement design.*

To elaborate the effect of genotypes on intercropping performance in terms of LER, the studies from maize-based were analyzed in detail. A total of 16 studies reported LER in maize-intercropping system and yielded 138 LER data records. The analysis shows that with the increasing number of maize genotypes included in the study, the LER range (maximum–minimum) increased significantly with an *R*^2^ of 0.58 (*p* = 0.00063) and 0.47 (*p* = 0.0046) in the regression of LER against number of genotype combinations, when the study of Santalla et al. (2001) that represents an outlier in terms of the number of genotypes combination tested (20 compared to 2–15) was included or excluded, respectively (Figure 5B).

### Mechanisms Underlying the Interactions Between Genotypes and Cropping System

In 20 out of the 69 studies, contrasting phenological or architectural characteristics of cereal and/or legume genotypes were highlighted, suggesting that the temporal and spatial differences among genotypes contributed to intercrop performance. These traits were broadly categorized into phenological and morphological traits (Table 6).

**TABLE 6:** Mechanisms of genotypes (G) complementarity in cereal/legume intercropping as mentioned in the consulted literature.

Cereal/legume	No. of G	Phenological and morphological traits that improve intercropping performance		References
		Cereals	Legumes	
Barley/pea	1 × 2		Long straw > short straw pea	Pappa et al., 2012
Barley/pea	5 × 6		Determinate > indeterminate pea	Hauggaard-Nielsen and Jensen, 2001
Barley/berseem clover	4 × 3	Early > late mature barley shorter > tall stature barley		Ross et al., 2004
Sorghum/groundnut	3 × 3	Late > early maturing sorghum when intercropped with early maturing groundnut	Late > early maturing groundnut when intercropped with early maturing sorghum	Tefera and Tana, 2002
Sorghum/cowpea	2 × 1	Short > tall stature sorghum		Odo, 1991
Sorghum/cowpea	4 × 4	Early > late mature sorghum		Rao and Willey, 1983
Rice/pigeon pea	2 × 2		Determinate > indeterminate pigeon pea	Ramakrishna and Ong, 1994
Millet/cowpea	2 × 2		Early > late mature cowpea	Ntare, 1990
Millet/cowpea	2 × 8		Early > early mature cowpea when intercropped with late mature millet	Ntare, 1989
Oat/faba bean	1 × 2		Indeterminate > determinate faba bean	Klimek-Kopyra et al., 2015
Oat/common vetch	3 × 3		Medium > late mature common vetch	Li et al., 2020
Oat/common vetch	4 × 1	Late > early mature oat and short > tall oat		Baxevanos et al., 2021
Wheat/faba bean	2 × 1	Tall > short straw of the oat		Haymes and Lee, 1999
Maize/cowpea	1 × 10		Early > late mature cowpea	Egbe et al., 2010
Maize/bean	2 × 7	Late > early mature of maize		Gebeyehu et al., 2006
Maize/bean	2 × 10	Short > tall maize		Davis and Garcia, 1983
Maize/common bean	1 × 2		Climbing > bushy bean	Clark and Francis, 1985
Maize/cowpea	3 × 2	Early > late mature maize		Ewansiha et al., 2014
Maize/bean	2 × 1	Short > tall maize		Munz et al., 2014
Maize/faba bean	1 × 3		Late > early mature faba bean	Fischer et al., 2020

*The empty cells are in the case no traits were mentioned. The first and second number in the second column (“No. of G”) refers to the number of genotypes on the first and of the second species mentioned in the first column (“Cereal/legume”).*

The phenological traits include growth duration (days to maturity, days required from emergence to flowering, and harvesting time), whereas morphological traits include shoot architecture (plant height) and growth habit (determinate/indeterminate growth) of the genotypes of each species. The reported phenological legume traits that affect intercropping, growth habit, and growth duration were reported more often than the morphological traits (long/short straw and climbing/bushy beans). However, no trend can be extracted from the provided information. In case of the cereals, only the phenological trait growth duration and the morphological trait plant height were reported. Three studies reported a better intercropping performance for early maturing cereals (maize, barley, and sorghum), whereas three others for late-maturing cereals (sorghum, oat, and maize). In case of plant height, five out of six studies reported improved intercropping performance for shorter cereal genotypes. Thus, besides a tendency for higher intercropping performance in case of short cereal genotypes, no conclusion can be drawn.

## Discussion

### Evaluation of the Performance of Different Cereal/Legume Species and Genotypes

The systematic assessment of LER from 35 independent studies showed the mean and median values of 1.26 and 1.24 (Figure 2A). This result is not far from the previously published meta-analysis result median values of 1.17 (Yu et al., 2015),1.16 (Yu et al., 2016), and 1.3 (Martin-Guay et al., 2018). These studies focus on the yield performance of crop species mixtures regardless of genotype. The median LER of 1.24 across 16 maize-based studies in our study is in line with a meta-analysis from 43 studies of maize/soybean of intercropping that reported an LER of 1.32 (Xu et al., 2020). Although the mean and median varied among different cereals, median LER was above one in all cereals.

The species and design effects were highly significant (*p* < 0.01) (Figure 4A), with a significant interaction (*p* < 0.05), mainly due to the higher LER of finger millet (1.66) compared to other species in additive designs. However, in replacement designs, no differences were observed among species. The overall LER was higher in additive designs compared to replacement designs. In an additive design, the planting density of both species in the mixture may be equivalent or somewhat reduced compared to their sole stand resulting in planting densities leading to density equivalent ratios > 1 and up to 2. For example, pea–oat mixtures may be composed of 100% peas and 20% oats compared to the pure stand densities (Gronle et al., 2015) or wheat–winter pea mixtures of 70% wheat with 50% pea (Timaeus et al., 2022). In replacement designs, the density of one sole crop species is proportionally (based on sole crop densities) replaced by the other species resulting in a density equivalent ratio of 1. For example, they may be composed of 50% barley and 50% pea compared to pure stand densities (Pappa et al., 2012). Although the planting proportion has an effect on LER, the range of effects depends on the species in the mixture because tillering in the case of cereals can compensate variable sowing densities (e.g., Finckh and Mundt, 1992; Finckh et al., 1999).

Compared to other cereal crops, millet was intercropped with short legumes, such as cowpea and pigeon pea. Intercropping the tall millet and sorghum cereals with shorter legumes permits better radiation use efficiency (Marshall and Willey, 1983; Matthews et al., 1991). Due to less resource competition by spatial segregation, yield in mixture and mono-cropping is comparable for both species which increased LER in additive compared to replacement designs. Nevertheless, a meta-analysis by Raseduzzaman and Jensen (2017) reported that in intercropping, replacement designs lead to higher yield stability compared to additive designs. The ANOVA across legume species (excluding faba bean, grass pea, guar and hairy vetch, berseem clover, and bitter vetch with *n* < 4 data points) resulted in significant differences. However, the effects of design and interaction were not significant (*p* > 0.05) (see Supplementary Table 5) with greater LER for pigeon pea and soybean compared to other legume species (see Supplementary Table 6). These two legume species are frequently intercropped with C4 cereals, such as maize, millet, and sorghum, which may increase the LER due to temporal niche differentiation (Yu et al., 2015; Xu et al., 2020).

The interaction between different cereal and legume genotypes and different cropping systems was significant in 75% of the studies that reported interaction effects of genotype × cropping system. This implies that in many studies, genotypes behave differently in sole vs. intercropping, often resulting in changes in the performance ranking of varieties between the sole crop and mixture (Woolley and Rodriguez, 1987; Baxevanos et al., 2017). The analyses of variation of different genotypes of cereal/legume intercrops within each selected study (Figure 5) revealed that the choice of the specific genotype combination could result in positive or negative yield effects compared to the median of all genotype combinations within each study. The largest LER range was found in a study with 20 different genotypes combinations (10 bean and two maize genotypes) (Santalla et al., 2001). This indicates the potential for high LER in case of appropriate genotype choice and highlights the potential for genotype or trait combination to optimize intercropping systems. However, this finding also emphasizes the need to develop a more general understanding of the mechanisms underlying these differences.

### Concept of Cereal/Legume Intercropping Niche Complementarity

Out of 20 studies assessing the mechanisms underlying the intercropping performance, 10 studies reported that intercropping performance was improved by cereal genotype, whereas the remaining 10 studies reported that the improvement was by legumes genotype. In some studies, however, a relatively high number of genotypes did not affect the intercropping performance. For instance, in the study of Hauggaard-Nielsen and Jensen (2001), none of the five barley genotypes affected LER, whereas pea genotype affected intercropping performance in terms of LER (Table 6).

In an intercropping system with annual species, the niche differentiation is a general mechanism underlying the yield advantage and better resource use efficiencies (Lithourgidis et al., 2011). Niche differentiation improves the use of resources according to species complementarity for light interception and the use of both soil mineral N and atmospheric N (Bedoussac et al., 2015). The selection of cereal and legume genotypes for better complementarity is important because the traits required for intercropping are those which enhance the complementary effects between the partners (Davis and Woolley, 1993). Niche differentiation among plant species occurs for the various environmental resources, such as light, water, and nutrient availability. It is driven by plant phenology and morphology that allows for partitioning of resources over time and space that facilitates coexistence (Silvertown, 2004). The trait differences in genotypes of cereals and legumes result in differences in phenology and morphology of the plants. Therefore, in cereal/legume mixtures, both species could have similar phenology but contrasting morphology or contrasting phenology and morphology, resulting in temporal and/or spatial niche complementarity (Gaudio et al., 2019). The contrasting characteristics of the genotypes play an important role in the complementarity of the species in intercropping (Hauggaard-Nielsen and Jensen, 2001; Gebeyehu et al., 2006).

The ecological niche separation concept describes the fact that different species involved may have different resource requirements at different times, as well as different sources of nutrition, e.g., root exploitation of top subsoil layers by one component vs. deeper exploitation by the other component, different growth patterns, or different affinities for the same nutrient (Malézieux et al., 2009). The temporal and spatial segregation of species in intercropping is useful in two ways: better resource capture, hence utilization of more resources, and enhanced resource use efficiency in a given unit of resource (Willey, 1990). The maturity rate and the growth habit of cereal and legumes define either the domination or suppression of one of the species in the mixture (Baxevanos et al., 2021). However, besides niche separation, additional mechanisms, such as mutual beneficial interactions *via* the soil microbiome, including biological N fixation, have to be considered (Hauggaard-Nielsen et al., 2009). Thus, in cereal legume mixtures, the contribution of biological N fixation through the leguminous partner is affected by the mineral N-supply level with strong effects on the competitive interactions and overall biological N fixation by the legume (Wang et al., 2015; Li et al., 2021).

#### Temporal Niche Complementarity of Cereal/Legume Intercropping

A trend for enhanced intercrop performance due to a specific trait related to phenology or temporal combination cannot be identified from the evaluated studies. Days required for maturity is one of the important factors for complementarity of species in intercropping. In this review, out of 20 studies reported that phenological and morphological traits affected intercropping performance with 12 studies indicated that the difference of days of maturity of different genotypes of cereals and/or legumes had an effect on the intercropping performance. However, it also varies in some cases, with a late-maturing genotype of either of the species meeting better the aim of cultivation compared to an early maturing genotype. In contrast, early genotypes could also be better compared to late maturing genotypes of one of the species (Table 6). In the study of Ntare (1989), intercropping an early maturing cowpea genotype with a relatively late-maturing millet genotype performed better by reducing the co-growth period to escape moisture scarcity and minimizing all components not affected equally in drought-prone areas. Another example of temporal complementarity is the combination of determinate field peas with a cereal where peas started maturing and releasing N from the roots around the time when the cereal flowers and requires increased N to fill its grains (Jensen et al., 2020; Timaeus et al., 2021b). The rate of development and time between sowing and harvesting of the components in intercropping provide the opportunity of temporally complementary use of incident radiation, thereby improving intercropping performance (Keating and Carberry, 1993). Tefera and Tana (2002) reported that the temporal niche complementarity of different genotypes in sorghum/groundnut intercropping influences the general performance of intercropping: partners that have a lower co-growth period produced higher yields compared to genotypes that have equal or higher co-growth period. Similar temporal niche complementarity was reported for millet/cowpea (Ntare, 1990), maize/cowpea systems (Egbe et al., 2010), and bean/maize systems (Gebeyehu et al., 2006). Depending on the aim of cultivation, the selection of cereal and legume genotypes with contrasting maturity periods will increase the intercropping yield advantage (Ross et al., 2004).

#### Spatial Niche Complementarity of Cereal/Legume Genotypes

Spatial niche complementarity can be exploited by the spatial arrangement of one component to maintain its full population, whereas allowing more space (and thus more resources) for another component (Willey, 1990). The spatial arrangement for better resource use efficiency could be classified as above-ground (canopy structure of both components) and below-ground (root system) (Gaudio et al., 2019). Canopy structure has considerable implications for intercropping systems. The erect open canopy of one component allows more transmission of radiation to shorter crops and enables more radiation use efficiency (Willey, 1990). The use of abiotic resources is improved according to species complementarity for light interception and the use of both soil mineral and atmospheric N.

In this review, 11 studies reported morphological differences of the genotype of either cereal or legumes to be involved in intercropping complementarity (Table 6). In most of these articles (7), plant height was observed. Whether the taller or the shorter genotype performed better varied. However, a tendency toward higher intercropping performance was observed with short cereal genotypes. Plant height and branching of long cycle pea genotypes varied between the sole and mixed cropping systems. This reveals the importance of the pea genotype choice in terms of morphology for intercropping systems (Barillot et al., 2014). The study by Hauggaard-Nielsen and Jensen (2001) revealed that pea genotypes with determinate growth absorbed more radiation under the barley canopy, which enhanced the intercropping performance compared to intercropping systems with indeterminate pea genotypes.

The growth habit of different genotypes of one species significantly affects the performance of other species, and thereby intercropping performance mainly by affecting radiation interception. Ramakrishna and Ong (1994) reported that the indeterminate pigeon pea genotype with indeterminate growth habit reduces the yield of rice by half due to the competitive advantage for radiation. In the barley/pea intercropping system, spatial complementarity due to pea genotypes has resulted in better N use efficiency of barley. An indeterminate pea genotype resulted in a greater proportion of peas in the intercrop yield due to high competitiveness, whereas a determinate pea genotype with normal leaves caused the highest degree of complimentary use of N sources by allowing barley to exploit the soil N sources efficiently, and they contribute with fixed N. However, indeterminate pea genotypes caused a reduced N uptake and yield of barley (Hauggaard-Nielsen and Jensen, 2001; Pappa et al., 2012). Based on the analyzed studies, we cannot draw a conclusion. In two articles, the intercropping performance was higher in case the growth of the legume partner was determinate, whereas in one study, it was higher for the indeterminate genotype.

### Gaps of Genotype and Trait Evaluation in Cereal/Legume Intercropping

Even though ample research reported on cereal legume intercropping, the number of publications that evaluated cereal/legume genotypes for complementarity in intercropping systems was very limited. Among the studies analyzed (69), only 20 (29%) articles indicate the contrasting traits of genotypes that contribute to intercropping performance. From those, the general mechanisms underlying the genotype cropping system were broadly classified as phenological and morphological heterogeneity of cereal and/or legume genotypes. However, in most of the studies, the contrasting characteristics of genotypes of either cereal or legumes and/or both of the species were not described well. The phenology of the crops has an impact on resource use over time (Gaudio et al., 2019). Consequently, cultivating genotypes with different phenological characteristics results in different temporal niche complementarity. The latter can increase the land use efficiencies, especially if N is released after grain filling of the legumes benefiting the cereals. Nevertheless, in most of the studies, sufficient information on phenology was not provided, and none of the studies reported the differences in the phenological stages of the genotypes.

Root growth and thus water and nutrient uptake are some of the most important factors in temporal and spatial heterogeneity (Hauggaard-Nielsen and Jen Yin et al., 2020). Root system distribution in time and space can partly explain competition. For instance, barley roots grow faster than pea roots (Hauggaard-Nielsen et al., 2001) and start nutrient acquisition earlier. Different genotypes of either the cereals or the legumes could have different root characteristics, which influence the competitive ability of the species. Streit et al. (2019) reported that mixtures of winter faba bean and winter wheat over yielded more below- than above-ground. The authors concluded that genotype differences in root biomass and over-yielding indicate the breeding potential of winter faba bean cultivars for mixed cropping. Legumes provide N to the agroecosystem through their exclusive capability to fix atmospheric N in a symbiotic relationship with soil rhizobia, but different genotypes of a legume species might have different capabilities in nodulation (Rodiño et al., 2011). Only a very limited number of studies considered the nutrient acquisition of different genotypes of cereals and legumes in intercropping. Different species have temporal niche differentiation in nutrient acquisition (Zhang et al., 2017). The symbiotic association of different legume genotypes and their rhizobia could also differ. The spatial complementarity of the genotypes in the nutrient acquisition is therefore important to increase the performance of intercropping. Hence, future research needs to address how different genotypes respond to nutrient competition, with a particular focus on below-ground traits.

Pest and disease resistance is one of the most important advantages of intercropping (Finckh et al., 2021). However, there are only a limited number of studies, which have considered genotype differences concerning pest and disease resistance in cereal/legume intercropping. Recent work has highlighted the importance of plant–plant interactions, either direct by mechanical, physical, or chemical cues, or mediated through soil/air microbiota, and the way they can affect plant immune system or other functions (Subrahmaniam et al., 2018; Khashi u Rahman et al., 2019; Zhu and Morel, 2019; Pélissier et al., 2021). Life cycle assessment (LCA) is a convenient, effective, and rarely used [but see Naudin et al. (2014)] approach for analyzing the environmental impact of cereal/legume intercropping, especially on the N cycle.

There are only a few studies considering the socio-economic importance of genotypes of both cereals and legumes species. Goshime et al. (2020) involved the farmers in the evaluation of genotypes. Different quality parameters of the genotypes not included in most of the articles hence could affect the acceptance of intercropping by farmers. The forage quality differences of legume genotypes were mostly ignored, and the number of studies on this topic is very limited. The consumer and market preference of different genotypes of cereals and/or legumes is also important in the selection of genotypes for intercropping. Therefore, in addition to morphological and phenological traits, other traits (roots, water and nutrient acquisition, and quality) and advantages in pest and weed suppression deserve attention to understand the mixing ability of different genotypes. Future research should consider pedigree analysis, functional genes, or key traits when selecting varieties tested in intercropping.

## Summary and Conclusion

We evaluated the observations of studies that included at least two genotypes of one species in cereal/legume intercropping. While the number of studies is inadequate for obtaining a comprehensive and reliable insight, our results point to the potential of genotype selection in intercropping, and future research should therefore emphasize genotype × cropping system interaction in cereal/legume intercropping. In total, the majority of the studies reported that there was a significant genotype–cropping system interaction revealing the importance of genotype selection for intercropping for more land productivity. Among the 69 analyzed studies, only 35 studies reported LER values. We determined a median LER of 1.24, which indicated that a combination of specific genotype cereals and legumes improves the land productivity by 24% on average. In addition, 85% of the LER data points of cereal/legume intercropping were greater than 1. On the other hand, 15% of the specific cereal/legume genotype combinations resulted in LER < 1 revealing that judicious choice of genotype combination in cereal/legume is indispensable.

Furthermore, the ANOVA across cereal species and design indicated that different species have different land-use efficiency in the different design types with finger millet having higher land-use efficiency than other crops in additive designs, whereas no difference was observed between the species in replacement designs. The number of studies, which report LER from different wheat genotypes, was very limited [but see Timaeus et al. (2022)]; because of the high importance of wheat for global food security, we suggest that more research is needed to investigate the performance of different wheat genotypes in intercropping. Conversely, the effect of design on land use efficiency in legumes is not significant, whereas species effect is significant. Temporal and spatial heterogeneity between the genotypes of the cereals and those of the legumes was mentioned in the selected studies as the main mechanism enhancing the overall performance of cereal–legume intercropping. However, the spatiotemporal heterogeneity of genotypes was not described sufficiently in most of the studies to allow a detailed analysis. Hence, future research studies should consider and report the genotypes’ traits more comprehensively, including root growth, soil nutrient and water acquisition, and diseases, among others. In most studies, only some agronomic traits of genotypes were emphasized ignoring other genotypic functional traits. Furthermore, we recommend that future research needs to evaluate a higher number of genotypes and their traits on various sites and under different climate and management conditions. It is impossible to test all possible combinations (genotype × genotype × environment × management) of intercropping in field trials. The complex interactions in intercropping can be disentangled by process-based agroecological models, which can help to identify the relevant influencing factors of intercrop performance. However, the prerequisite is an understanding of the basic mechanisms.

## Author Contributions

All authors listed have made a substantial, direct, and intellectual contribution to the work, and approved it for publication.

## Conflict of Interest

The authors declare that the research was conducted in the absence of any commercial or financial relationships that could be construed as a potential conflict of interest.

## Publisher’s Note

All claims expressed in this article are solely those of the authors and do not necessarily represent those of their affiliated organizations, or those of the publisher, the editors and the reviewers. Any product that may be evaluated in this article, or claim that may be made by its manufacturer, is not guaranteed or endorsed by the publisher.
