# Supplementary material for: Mixture × Genotype Effects in Cereal/Legume Intercropping
Source: Front Plant Sci. 2022 Apr 1;13:846720. doi: 10.3389/fpls.2022.846720 (PMC9011192; doi:10.3389/fpls.2022.846720)
Supplement: Supplementary file 1 [file Data_Sheet_1.docx]

***Supplementary Material***

**Table S1**. Detailed list of publication search terms

Cereals genotype evaluation intercropping, Cereals genotype evaluation mixed cropping, Cereals genotype evaluation mixture,

Legumes genotype evaluation intercropping, Legumes genotype evaluation mixed cropping, Legumes genotype evaluation mixture,

Barley genotype evaluation intercropping, barley genotype evaluation mixed cropping, barley genotype evaluation mixture,

*Hordeum vulgare* genotype evaluation intercropping, *Hordeum vulgare* genotype evaluation mixed cropping, *Hordeum vulgare* genotype evaluation mixture,

 Maize genotype evaluation intercropping, Maize genotype evaluation mixed cropping, Maize genotype evaluation mixture,

*zea mays* genotype evaluation intercropping, *zea mays* genotype evaluation mixed cropping, *zea mays* genotype evaluation mixture,

 Wheat genotype evaluation intercropping, wheat genotype evaluation mixed cropping, wheat genotype evaluation mixture,

*Triticum aestivum* genotype evaluation intercropping, *Triticum aestivum* genotype evaluation mixed cropping, *Triticum aestivum* genotype evaluation mixture,

Oat genotype evaluation intercropping, Oat genotype evaluation mixed cropping, Oat genotype evaluation mixture,

*Avena sativa* genotype evaluation intercropping, *Avena sativa* genotype evaluation mixed cropping, *Avena sativa* genotype evaluation mixture,

Sorghum genotype evaluation intercropping, sorghum genotype evaluation mixed cropping, sorghum genotype evaluation mixture,

*Sorghum bicolor* genotype evaluation intercropping, *Sorghum bicolor* genotype evaluation mixed cropping, *Sorghum bicolor* genotype evaluation mixture,

Millet genotype evaluation intercropping, millet genotype evaluation mixed cropping, millet genotype evaluation mixture,

(*Eleusine coracana*) genotype evaluation intercropping, (*Eleusine coracana*) genotype evaluation mixed cropping, (*Eleusine coracana*) genotype evaluation mixture

**Table S2.** Countries of cereal/legume experiment conducted

| Country | No. of studies | Country | No. of studies |
| --- | --- | --- | --- |
| Australia | 1 | Ghana | 1 |
| Burkina Faso | 1 | Greece | 1 |
| Canada | 4 | India | 4 |
| China | 6 | Iran | 2 |
| Colombia | 2 | Italy | 1 |
| Costa Rica | 1 | Ivory Coast | 1 |
| Côte d’Ivoire | 1 | Kenya | 2 |
| Denmark | 2 | Niger | 3 |
| Egypt | 2 | Nigeria | 6 |
| UK | 4 | Poland | 1 |
| Ethiopia | 5 | South Africa | 1 |
| Finland | 1 | Spain | 4 |
| France | 2 | Tanzania | 2 |
| Germany | 3 | USA | 5 |

**Table S3**. Analysis of variance of LER across cereal species and design

Source DF Sum of Square Mean Square F Value Pr(> F)

Cereal 3 0.7459 0.2486 3.97 0.0089

Design 1 1.1171 1.1171 17.82 0.0000

Cereal:Design 3 0.5668 0.1889 3.01 0.0311

Error 208 13.0425 0.0627

Total 215 15.4724

**Table S4.** Pairwise Mean Comparison of cereal species within each two designs. Tukeys's Honest Significant Difference (HSD) Test, Alpha = 0.05

Cereals design = additive means: group design = replacement means: group

maize 1.2759 b 1.1872 a

millet 1.6675 a 1.2326 a

oat 1.3411 b 1.1266 a

sorghum 1.4124 b 1.2560 a

**Table S5**. Analysis of variance of LER across legume species and design

Source DF Sum of Square Mean Square F Value Pr(> F)

Legumes 6 5.2348 0.8725 18.09 0.0000

Design 1 0.0000 0.0000 0.00 0.9867

Legumes:Design 5 0.3626 0.0725 1.50 0.1894

Error 227 10.9450 0.0482

Total 239 16.5425

**Table S6.** Pairwise Mean Comparison of legumes species Tukeys's Honest Significant Difference (HSD) Test, Alpha = 0.05

**Legumes means group**

common bean 1.19 b

common vetch 1.24 b

cowpea 1.17 b

groundnut 1.24 b

pea 1.13 b

pigeon pea 1.48 a

soybean 1.55 a
